# *rac*-1-(5-Bromo-2-hy­droxy­phen­yl)-1-oxopropan-2-yl morpholine-4-carbo­dithio­ate

**DOI:** 10.1107/S1600536813017509

**Published:** 2013-06-29

**Authors:** Laura G. Sarbu, Cristian G. Hrib, Lucian M. Birsa

**Affiliations:** a Department of Chemistry, "Al. I. Cuza" University Iasi, 11 Carol I Bvd, Iasi 700506, Romania; b Chemisches Institut der Otto-von-Guericke-Universität, Universitätsplatz 2, D-39116 Magdeburg, Germany

## Abstract

In the racemic title compound, C_14_H_16_BrNO_3_S_2_, synthesized from the corresponding ω-bromo­propio­phenone, the dihedral angle between the plane of the phenol group and that of the planar section [maximum deviation = 0.040 (2) Å] of the morpholine-4-carbodi­thiol­ate moiety is 76.36 (10)°. A strong intra­molecular phenol O—H⋯O hydrogen bond if present in the mol­ecule. In the crystal, only weak C—H⋯S and C—H⋯O inter­actions are found.

## Related literature

For the synthesis and applications of di­thio­carbamates, see: Buu-Hoi & Lavit (1955 ▶); WHO (1998 ▶). For applications of 1,3-di­thiol­ium salts, see: Narita & Pittman (1976 ▶); Birsa & Asaftei (2008 ▶). For the structure of a related morpholine-4-carbodi­thio­ate, see: Bahrin *et al.* (2012 ▶).
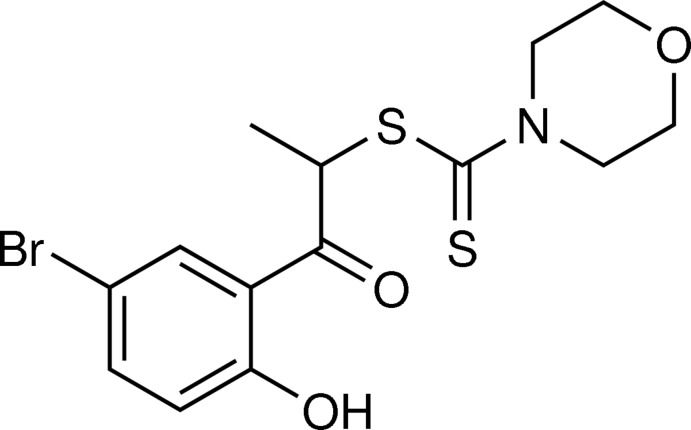


## Experimental

### 

#### Crystal data

C_14_H_16_BrNO_3_S_2_
*M* *_r_* = 390.31
Monoclinic, 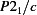

*a* = 11.182 (2) Å
*b* = 19.660 (4) Å
*c* = 7.4593 (15) Å
β = 105.44 (3)°
*V* = 1580.6 (5) Å^3^
*Z* = 4
Mo *K*α radiation
μ = 2.87 mm^−1^
*T* = 153 K
0.54 × 0.48 × 0.30 mm

#### Data collection

Stoe IPDS 2T area-detector diffractometer
Absorption correction: for a sphere [modification of the interpolation procedure of Dwiggins (1975 ▶)] *T*_min_ = 0.114, *T*_max_ = 0.140
17019 measured reflections
4246 independent reflections
3807 reflections with *I* > 2σ(*I*)
*R*_int_ = 0.072

#### Refinement

*R*[*F* ^2^ > 2σ(*F* ^2^)] = 0.045
*wR*(*F* ^2^) = 0.102
*S* = 1.16
4246 reflections
195 parameters
H atoms treated by a mixture of independent and constrained refinement
Δρ_max_ = 0.71 e Å^−3^
Δρ_min_ = −0.78 e Å^−3^

### 

Data collection: *X-AREA* (Stoe & Cie, 2002 ▶); cell refinement: *X-AREA*; data reduction: *X-RED* (Stoe & Cie, 2002 ▶); program(s) used to solve structure: *SHELXS97* (Sheldrick, 2008 ▶); program(s) used to refine structure: *SHELXL97* (Sheldrick, 2008 ▶); molecular graphics: *XP* in *SHELXTL* (Sheldrick, 2008 ▶); software used to prepare material for publication: *SHELXL97*.

## Supplementary Material

Crystal structure: contains datablock(s) I, global. DOI: 10.1107/S1600536813017509/zs2265sup1.cif

Structure factors: contains datablock(s) I. DOI: 10.1107/S1600536813017509/zs2265Isup2.hkl

Supplementary material file. DOI: 10.1107/S1600536813017509/zs2265Isup3.cml

Additional supplementary materials:  crystallographic information; 3D view; checkCIF report

## Figures and Tables

**Table 1:** Hydrogen-bond geometry (Å, °)

*D*—H⋯*A*	*D*—H	H⋯*A*	*D*⋯*A*	*D*—H⋯*A*
O1—H1⋯O2	0.76 (4)	1.86 (4)	2.558 (3)	151 (5)
C4—H4⋯S2^i^	0.95	2.79	3.712 (3)	164
C3—H3⋯O3^ii^	0.95	2.51	3.443 (4)	168
C12—H12*B*⋯O1^iii^	0.99	2.46	3.454 (4)	178

Symmetry codes: (i) 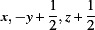; (ii) 
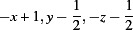
; (iii) 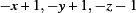.

**Fig. 1.**
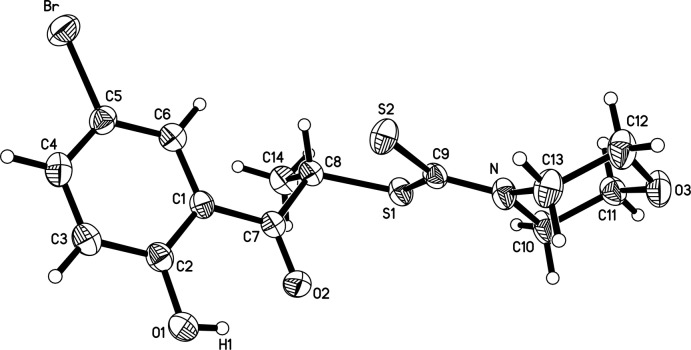
Molecular conformation and atom numbering scheme for the title compound, with thermal ellipsoids drawn at the 50% probability level.

## supplementary crystallographic information

### Comment

Dithiocarbamates have important uses as chemical precursors, effluent additives, agricultural pesticides, and in experimental and clinical medicine (WHO, 1998). In particular, phenacyldithiocarbamates are important precursors of 1,3-dithiolium salts (Birsa & Asaftei, 2008), which in turn are well known precursors of tetrathiafulvalenes (Narita & Pittman, 1976). The racemic title compound C_14_H_16_BrNO_3_S_2_ has been synthesized by the reaction of 2-bromo-1-(5-bromo-2-hydroxyphenyl)-propan-1-one (Buu-Hoi & Lavit, 1955) with a salt of morpholine-4-carbodithioate. In this compound (Fig. 1), the dihedral angle between the phenolic ring system and the plane defined by atoms S1,S2,C9,C10,C13 of the morpholine-4-carbodithiolate moiety is 76.36 (10)°. The maximum deviation from the least-squares plane to this fragment is 0.040 (2) Å (C9). A strong intramolecular hydrogen bond between the phenolic O1—H group and a carbonyl O-atom acceptor atom of the side chain (O2) is present (Table 1). In the crystal there is a weak intermolecular C4—H···S2^i^ association [3.712 (3)Å] and weak C3—H···O3^ii^ and C12—H···O1^iii^ hydrogen bonds [3.443 (4) and 3.454(4 Å, respectively] (for symmetry codes, see Table 1).

### Experimental

To a solution of 0.924 g (3 mmol) 2-bromo-1-(5-bromo-2-hydroxyphenyl)-propan-1-one (Buu-Hoi & Lavit, 1955) in 10 ml of acetone was added a solution of 0.75 g (3 mmol) morpholinium morpholine-4-carbodithioate in 10 ml acetone-water (1:1). The reaction mixture was heated at reflux for 10 min, cooled to room temperature and then poured into water. The precipitate was filtered, washed with water and dried (m.p. 412–413 K). IR (ATR): ν_max_ 2852, 1643, 1466, 1424, 1258, 1228, 1111, 999, 624, 543 cm^-1^. ^1^H NMR (300 MHz, DMSO-d6): *δ* = 1.57 (d, 3H, CH3), 3.74 (m, 4H, 2CH2-O), 4.09 (m, 4H, 2CH2-N), 5.75 (q, 1H, CH), 6.88 (d, 3 J=8.0 Hz, 1H), 7.53 (dd, 3 J=8.0 Hz, 4 J=1.1 Hz, 1H), 8.04 (d, 4 J=1.1 Hz, 1H), 11.04 (s, 1H, OH). ^13^C{^1^H} NMR (75 MHz, DMSO-d6): *δ* = 17.2 (*q*), 51.2 (*d*), 52.3 (*t*), 66.7 (*t*), 111.3 (*s*), 119.8 (*d*), 121.1 (*s*), 133.0 (*d*), 133.3 (*d*), 162.4 (*s*), 194.4 (*s*), 203.5 (*s*).

### Refinement

The C-bound H-atoms were included at calculated positions and treated using a riding model, with aromatic C—H = 0.95 Å, methylene C—H = 0.99 Å and methine C—H = 1.00 Å and *U*_iso_(H) = 1.2*U*_eq_(C), or with methyl C—H = 0.98 Å and *U*_iso_(H) = 1.5*U*_eq_(C). The phenolic H-atom (H1) was free refined.

### Crystal data

**Table:**

C_14_H_16_BrNO_3_S_2_	*F*(000) = 792
*M**_r_* = 390.31	*D*_x_ = 1.640 Mg m^−^^3^
Monoclinic, *P*2_1_/*c*	Melting point = 412–413 K
Hall symbol: -P 2ybc	Mo *K*α radiation, λ = 0.71073 Å
*a* = 11.182 (2) Å	Cell parameters from 26120 reflections
*b* = 19.660 (4) Å	θ = 2.2–29.7°
*c* = 7.4593 (15) Å	µ = 2.87 mm^−^^1^
β = 105.44 (3)°	*T* = 153 K
*V* = 1580.6 (5) Å^3^	Prism, colourless
*Z* = 4	0.54 × 0.48 × 0.30 mm

### Data collection

**Table:**

Stoe IPDS 2T area-detector diffractometer	4246 independent reflections
Radiation source: fine-focus sealed tube	3807 reflections with *I* > 2σ(*I*)
Graphite monochromator	*R*_int_ = 0.072
Detector resolution: 6.67 pixels mm^-1^	θ_max_ = 29.2°, θ_min_ = 2.8°
rotation method scans	*h* = −15→13
Absorption correction: for a sphere [modification of the interpolation procedure of Dwiggins (1975)]	*k* = −25→26
*T*_min_ = 0.114, *T*_max_ = 0.140	*l* = −10→10
17019 measured reflections	

### Refinement

**Table:**

Refinement on *F*^2^	Primary atom site location: structure-invariant direct methods
Least-squares matrix: full	Secondary atom site location: difference Fourier map
*R*[*F*^2^ > 2σ(*F*^2^)] = 0.045	Hydrogen site location: inferred from neighbouring sites
*wR*(*F*^2^) = 0.102	H atoms treated by a mixture of independent and constrained refinement
*S* = 1.16	*w* = 1/[σ^2^(*F*_o_^2^) + (0.0355*P*)^2^ + 1.2363*P*] where *P* = (*F*_o_^2^ + 2*F*_c_^2^)/3
4246 reflections	(Δ/σ)_max_ = 0.001
195 parameters	Δρ_max_ = 0.71 e Å^−^^3^
0 restraints	Δρ_min_ = −0.78 e Å^−^^3^

### Special details

**Table:**

Experimental. Absorption correction: interpolation using International Tables Vol C, Table 6.3.3.3 for values of muR in the range 0-2.5, and International Tables Vol. II, Table 5.3.6 B for µR in the range 2.6-10.0. The interpolation procedure (Dwiggins, 1975) was used with some modification.
Geometry. All e.s.d.'s (except the e.s.d. in the dihedral angle between two l.s. planes) are estimated using the full covariance matrix. The cell e.s.d.'s are taken into account individually in the estimation of e.s.d.'s in distances, angles and torsion angles; correlations between e.s.d.'s in cell parameters are only used when they are defined by crystal symmetry. An approximate (isotropic) treatment of cell e.s.d.'s is used for estimating e.s.d.'s involving l.s. planes.
Refinement. Refinement of *F*^2^ against ALL reflections. The weighted *R*-factor *wR* and goodness of fit *S* are based on *F*^2^, conventional *R*-factors *R* are based on *F*, with *F* set to zero for negative *F*^2^. The threshold expression of *F*^2^ > σ(*F*^2^) is used only for calculating *R*-factors(gt) *etc*. and is not relevant to the choice of reflections for refinement. *R*-factors based on *F*^2^ are statistically about twice as large as those based on *F*, and *R*- factors based on ALL data will be even larger.

### Fractional atomic coordinates and isotropic or equivalent isotropic displacement parameters (Å^2^)

**Table:**

	*x*	*y*	*z*	*U*_iso_*/*U*_eq_	
N	0.7046 (2)	0.59260 (10)	−0.3669 (3)	0.0291 (4)	
Br	0.94733 (3)	0.200387 (14)	0.15953 (5)	0.04007 (10)	
S1	0.82823 (6)	0.55807 (3)	−0.03082 (8)	0.02727 (13)	
S2	0.72078 (6)	0.45824 (3)	−0.33614 (9)	0.03161 (15)	
O1	0.50712 (18)	0.37914 (11)	0.1313 (3)	0.0344 (4)	
H1	0.525 (4)	0.416 (2)	0.120 (6)	0.059 (13)*	
O2	0.63809 (17)	0.48119 (9)	0.0864 (3)	0.0316 (4)	
O3	0.6883 (2)	0.70107 (9)	−0.6200 (3)	0.0378 (5)	
C1	0.7188 (2)	0.36994 (12)	0.1107 (3)	0.0243 (4)	
C2	0.6083 (2)	0.34177 (13)	0.1327 (3)	0.0269 (5)	
C3	0.5990 (3)	0.27144 (14)	0.1556 (4)	0.0320 (5)	
H3	0.5233	0.2522	0.1665	0.038*	
C4	0.6994 (3)	0.23020 (13)	0.1624 (4)	0.0320 (5)	
H4	0.6934	0.1826	0.1794	0.038*	
C5	0.8095 (2)	0.25820 (12)	0.1445 (3)	0.0281 (5)	
C6	0.8199 (2)	0.32679 (13)	0.1174 (3)	0.0269 (5)	
H6	0.8955	0.3450	0.1031	0.032*	
C7	0.7261 (2)	0.44422 (12)	0.0859 (3)	0.0246 (4)	
C8	0.8494 (2)	0.47533 (12)	0.0777 (3)	0.0255 (4)	
H8	0.8923	0.4444	0.0084	0.031*	
C9	0.7432 (2)	0.53841 (11)	−0.2621 (3)	0.0238 (4)	
C10	0.7202 (3)	0.66338 (12)	−0.3028 (4)	0.0359 (6)	
H10A	0.7798	0.6655	−0.1782	0.043*	
H10B	0.6397	0.6816	−0.2927	0.043*	
C11	0.7672 (3)	0.70563 (12)	−0.4379 (4)	0.0311 (5)	
H11A	0.7733	0.7537	−0.3972	0.037*	
H11B	0.8513	0.6900	−0.4379	0.037*	
C12	0.6805 (3)	0.63236 (14)	−0.6823 (4)	0.0393 (7)	
H12A	0.7643	0.6159	−0.6817	0.047*	
H12B	0.6274	0.6299	−0.8116	0.047*	
C13	0.6270 (3)	0.58721 (13)	−0.5588 (4)	0.0353 (6)	
H13A	0.5411	0.6015	−0.5652	0.042*	
H13B	0.6249	0.5394	−0.6016	0.042*	
C14	0.9305 (3)	0.48568 (15)	0.2771 (4)	0.0332 (5)	
H14A	0.8848	0.5126	0.3476	0.050*	
H14B	1.0067	0.5097	0.2740	0.050*	
H14C	0.9517	0.4413	0.3372	0.050*	

### Atomic displacement parameters (Å^2^)

**Table:**

	*U*^11^	*U*^22^	*U*^33^	*U*^12^	*U*^13^	*U*^23^
N	0.0470 (13)	0.0183 (9)	0.0238 (9)	−0.0032 (8)	0.0125 (9)	−0.0015 (7)
Br	0.03949 (16)	0.03294 (15)	0.04765 (17)	0.00946 (11)	0.01139 (12)	0.00128 (11)
S1	0.0348 (3)	0.0231 (3)	0.0248 (3)	−0.0061 (2)	0.0095 (2)	−0.0022 (2)
S2	0.0381 (3)	0.0189 (2)	0.0340 (3)	−0.0042 (2)	0.0029 (3)	−0.0027 (2)
O1	0.0267 (9)	0.0330 (10)	0.0462 (11)	−0.0017 (7)	0.0143 (8)	0.0047 (8)
O2	0.0280 (9)	0.0295 (9)	0.0393 (10)	0.0013 (7)	0.0127 (8)	0.0029 (7)
O3	0.0579 (13)	0.0268 (9)	0.0293 (9)	0.0021 (8)	0.0125 (9)	0.0056 (7)
C1	0.0268 (11)	0.0257 (10)	0.0202 (9)	−0.0017 (8)	0.0058 (8)	0.0018 (8)
C2	0.0256 (11)	0.0304 (11)	0.0241 (10)	−0.0029 (9)	0.0056 (9)	0.0017 (9)
C3	0.0311 (12)	0.0326 (12)	0.0329 (12)	−0.0058 (10)	0.0096 (10)	0.0015 (10)
C4	0.0391 (14)	0.0263 (11)	0.0300 (12)	−0.0037 (10)	0.0080 (11)	0.0019 (9)
C5	0.0326 (12)	0.0269 (11)	0.0242 (11)	0.0037 (9)	0.0063 (9)	0.0013 (9)
C6	0.0257 (11)	0.0294 (11)	0.0245 (10)	−0.0014 (9)	0.0049 (9)	−0.0002 (9)
C7	0.0247 (11)	0.0260 (10)	0.0232 (10)	−0.0024 (8)	0.0067 (8)	0.0014 (8)
C8	0.0254 (11)	0.0263 (10)	0.0256 (10)	−0.0020 (9)	0.0082 (9)	0.0013 (8)
C9	0.0271 (11)	0.0206 (9)	0.0263 (10)	−0.0040 (8)	0.0117 (9)	−0.0005 (8)
C10	0.0671 (19)	0.0182 (10)	0.0289 (12)	−0.0029 (11)	0.0240 (13)	0.0000 (9)
C11	0.0418 (14)	0.0247 (11)	0.0306 (12)	−0.0031 (10)	0.0161 (11)	−0.0006 (9)
C12	0.062 (2)	0.0291 (12)	0.0234 (11)	0.0014 (12)	0.0061 (12)	−0.0007 (9)
C13	0.0399 (14)	0.0272 (12)	0.0335 (13)	−0.0032 (10)	0.0006 (11)	0.0011 (10)
C14	0.0287 (12)	0.0398 (14)	0.0292 (11)	−0.0039 (10)	0.0044 (10)	0.0007 (10)

### Geometric parameters (Å, º)

**Table:**

N—C9	1.324 (3)	C4—H4	0.9500
N—C10	1.467 (3)	C5—C6	1.373 (3)
N—C13	1.467 (3)	C6—H6	0.9500
Br—C5	1.894 (3)	C7—C8	1.524 (3)
S1—C9	1.776 (2)	C8—C14	1.536 (3)
S1—C8	1.804 (2)	C8—H8	1.0000
S2—C9	1.667 (2)	C10—C11	1.504 (3)
O1—C2	1.346 (3)	C10—H10A	0.9900
O1—H1	0.77 (4)	C10—H10B	0.9900
O2—C7	1.224 (3)	C11—H11A	0.9900
O3—C11	1.412 (3)	C11—H11B	0.9900
O3—C12	1.424 (3)	C12—C13	1.512 (4)
C1—C6	1.403 (3)	C12—H12A	0.9900
C1—C2	1.404 (3)	C12—H12B	0.9900
C1—C7	1.477 (3)	C13—H13A	0.9900
C2—C3	1.400 (4)	C13—H13B	0.9900
C3—C4	1.375 (4)	C14—H14A	0.9800
C3—H3	0.9500	C14—H14B	0.9800
C4—C5	1.387 (4)	C14—H14C	0.9800
			
C9—N—C10	125.4 (2)	N—C9—S2	124.63 (19)
C9—N—C13	122.2 (2)	N—C9—S1	113.86 (17)
C10—N—C13	112.0 (2)	S2—C9—S1	121.47 (14)
C9—S1—C8	102.22 (11)	N—C10—C11	109.7 (2)
C2—O1—H1	106 (3)	N—C10—H10A	109.7
C11—O3—C12	110.0 (2)	C11—C10—H10A	109.7
C6—C1—C2	118.9 (2)	N—C10—H10B	109.7
C6—C1—C7	122.1 (2)	C11—C10—H10B	109.7
C2—C1—C7	118.9 (2)	H10A—C10—H10B	108.2
O1—C2—C3	116.8 (2)	O3—C11—C10	111.7 (2)
O1—C2—C1	123.2 (2)	O3—C11—H11A	109.3
C3—C2—C1	120.0 (2)	C10—C11—H11A	109.3
C4—C3—C2	120.0 (2)	O3—C11—H11B	109.3
C4—C3—H3	120.0	C10—C11—H11B	109.3
C2—C3—H3	120.0	H11A—C11—H11B	107.9
C3—C4—C5	120.0 (2)	O3—C12—C13	111.0 (2)
C3—C4—H4	120.0	O3—C12—H12A	109.4
C5—C4—H4	120.0	C13—C12—H12A	109.4
C6—C5—C4	121.1 (2)	O3—C12—H12B	109.4
C6—C5—Br	119.9 (2)	C13—C12—H12B	109.4
C4—C5—Br	119.03 (19)	H12A—C12—H12B	108.0
C5—C6—C1	120.0 (2)	N—C13—C12	109.0 (2)
C5—C6—H6	120.0	N—C13—H13A	109.9
C1—C6—H6	120.0	C12—C13—H13A	109.9
O2—C7—C1	121.1 (2)	N—C13—H13B	109.9
O2—C7—C8	119.9 (2)	C12—C13—H13B	109.9
C1—C7—C8	118.8 (2)	H13A—C13—H13B	108.3
C7—C8—C14	108.8 (2)	C8—C14—H14A	109.5
C7—C8—S1	111.58 (17)	C8—C14—H14B	109.5
C14—C8—S1	106.75 (17)	H14A—C14—H14B	109.5
C7—C8—H8	109.9	C8—C14—H14C	109.5
C14—C8—H8	109.9	H14A—C14—H14C	109.5
S1—C8—H8	109.9	H14B—C14—H14C	109.5
			
C6—C1—C2—O1	178.9 (2)	O2—C7—C8—S1	−25.7 (3)
C7—C1—C2—O1	0.4 (3)	C1—C7—C8—S1	159.95 (17)
C6—C1—C2—C3	−1.8 (3)	C9—S1—C8—C7	−65.12 (19)
C7—C1—C2—C3	179.7 (2)	C9—S1—C8—C14	176.19 (17)
O1—C2—C3—C4	−178.6 (2)	C10—N—C9—S2	178.1 (2)
C1—C2—C3—C4	2.1 (4)	C13—N—C9—S2	5.6 (4)
C2—C3—C4—C5	−0.7 (4)	C10—N—C9—S1	−4.1 (3)
C3—C4—C5—C6	−0.9 (4)	C13—N—C9—S1	−176.5 (2)
C3—C4—C5—Br	178.7 (2)	C8—S1—C9—N	173.43 (19)
C4—C5—C6—C1	1.2 (4)	C8—S1—C9—S2	−8.64 (18)
Br—C5—C6—C1	−178.47 (18)	C9—N—C10—C11	133.4 (3)
C2—C1—C6—C5	0.2 (3)	C13—N—C10—C11	−53.5 (3)
C7—C1—C6—C5	178.6 (2)	C12—O3—C11—C10	−60.2 (3)
C6—C1—C7—O2	−177.6 (2)	N—C10—C11—O3	56.1 (3)
C2—C1—C7—O2	0.8 (3)	C11—O3—C12—C13	61.1 (3)
C6—C1—C7—C8	−3.3 (3)	C9—N—C13—C12	−132.4 (3)
C2—C1—C7—C8	175.1 (2)	C10—N—C13—C12	54.3 (3)
O2—C7—C8—C14	91.8 (3)	O3—C12—C13—N	−57.7 (3)
C1—C7—C8—C14	−82.6 (3)		

### Hydrogen-bond geometry (Å, º)

**Table:**

*D*—H···*A*	*D*—H	H···*A*	*D*···*A*	*D*—H···*A*
O1—H1···O2	0.76 (4)	1.86 (4)	2.558 (3)	151 (5)
C4—H4···S2^i^	0.95	2.79	3.712 (3)	164
C3—H3···O3^ii^	0.95	2.51	3.443 (4)	168
C12—H12*B*···O1^iii^	0.99	2.46	3.454 (4)	178

Symmetry codes: (i) *x*, −*y*+1/2, *z*+1/2; (ii) −*x*+1, *y*−1/2, −*z*−1/2; (iii) −*x*+1, −*y*+1, −*z*−1.
